# Determining the interconnection between personality and physical activity on perceived stress in a diverse sample

**DOI:** 10.3389/fpsyg.2025.1502890

**Published:** 2025-10-24

**Authors:** Rena Elizabeth Courtney, Mary Josephine Schadegg, Patrick Brice, Bruce H. Friedman, Jason Fanning

**Affiliations:** ^1^PREVAIL Center for Chronic Pain, Salem VA Health Care System, Salem, VA, United States; ^2^Department of Psychiatry and Behavioral Medicine, Virginia Tech Carilion School of Medicine, Roanoke, VA, United States; ^3^Veterans Integrated Service Network 6 Mental Illness Research, Education, and Clinical Center (VISN 6 MIRECC), Durham, NC, United States; ^4^Department of Psychiatry, NYU Grossman School of Medicine, New York, NY, United States; ^5^Department of Psychology, Gallaudet University, Washington, DC, United States; ^6^Department of Psychology, Virginia Tech, Blacksburg, VA, United States; ^7^Department of Health and Exercise Science, Wake Forest University, Winston-Salem, NC, United States

**Keywords:** exercise, individual differences, precision medicine, mental health, personality

## Abstract

**Introduction:**

Stress is considered an epidemic with far-reaching deleterious impacts across multiple domains while engagement in physical activity (PA), a proven way to reduce perceived stress (PS), remains low. The purpose of this study was to determine whether the Five Factor Model (FFM) personality factors and self-reported PA have independent main effects on PS, and to examine the interaction between PA and personality on PS. We predicted that neuroticism, extraversion, conscientiousness, and PA would predict PS.

**Methods:**

A cross-sectional study was conducted. Participants were adults without anxiety disorders who completed online surveys including the IPIP-NEO-120, PSS-10, and IPAQ-Long. An iterative series of linear regressions were used to determine whether personality characteristics and PA were related to PS and to detect interactive effects.

**Results:**

The final sample included 276 participants who were, on average, mostly non-White (62%), reported moderate level of PS (Mean PSS Total Score = 17.01, SD = 6.74), and reported high levels of PA (Mean Total MET-minutes per week = 2,994.81, SD = 2,620.92). The hypothesis was partially supported such that neuroticism (*B* = 0.02, *p* = <0.001) and conscientiousness (*B* = 0.02, *p* = <0.001) predicted PS, though PA did not. Only openness to experience demonstrated an interactive effect, with those high in openness to experience and PA tended to have higher PS.

**Discussion:**

This study provides additional support for the relationship between the FFM of personality and PS. Future studies on the connection between PS, personality, and PA may benefit from the use of a combined approach, including both self-report and objective measures of PA.

## Introduction

Chronic stress, defined as stress resulting from an “eliciting stimulus that remains in the environment for an extended period of time” (p. 28) (Miller et al., 2007), is considered an epidemic in modern society (Newbegin, 2014; Siervo et al., 2009). This epidemic results (Miller et al., 2007) in numerous long-term negative implications for mental and physical health, quality of life, and all-cause mortality (Da Estrela et al., 2021; Rodgers et al., 2021; Rohleder, 2019; Yaribeygi et al., 2017). Specifically, the presence of stress has been linked to the progression, onset, and severity of several long-term health outcomes including obesity, cardiovascular disease, cancer, insomnia, pain, anxiety, depression, executive and cognitive dysfunction, and death, including death by suicide (Chrousos, 2009; Cohen et al., 2007; Dai et al., 2020; Gallagher et al., 2018; Lagraauw et al., 2015; Østerås et al., 2015; Kanani and Sheikh, 2024).

Physical activity (PA), which is considered any bodily movement that expends energy above a basal level (U.S. Department of Health and Human Services, 2018), has emerged as an effective prevention and treatment strategy for perceived stress (PS). Current guidelines recommend at least 150 min of moderate PA or 75 min of vigorous PA and muscle strengthening exercises at least 2 days per week for optimum benefit, including reduced PS (U.S. Department of Health and Human Services, 2018). Engagement in these recommended levels of PA, regardless of the domain (e.g., biking, walking, swimming, running) is generally regarded as a useful lifestyle approach to stress management (Franklin et al., 2021), with benefits for both physical (Kaminsky et al., 2022; Viña et al., 2012) and mental (DeBoer et al., 2012; De la Rosa et al., 2020; Salmon, 2001) health, and a decreased risk for mortality (Zhao et al., 2020). Thus, manualized treatments that aim to increase engagement in PA, thereby reducing PS and improving mental health disorders, have become widely available. However, the relationship between PA and PS is not always linear (Salmon, 2001) and few people engage in the recommended levels of PA long-term (Evenson et al., 2015; Yang et al., 2019). Furthermore, emerging evidence suggests that many individuals do not in fact experience improvements in affect or PS *during* an activity bout, and factors such as activity intensity can contribute to worsening feelings of PS or aversion (Shimura et al., 2023; Petruzzello et al., 1991; Box and Petruzzello, 2020). The complex relationship between PA and affect involves several factors such as autonomy, weight, anxiety sensitivity, fitness level, fitness phobia, and exercise intensity which may increase negative affect, including PS (Ekkekakis and Lind, 2006; Ekkekakis et al., 2010; Ekkekakis et al., 2005). Given PS’ prevalence and far-reaching deleterious impacts, as well as the heterogeneous impact of PA on affect, understanding individual differences that would improve the impact of PA on PS are vital. One of these individual differences may be personality. After all, the same stressor may cause a variety of reactions that vary in duration, expression, and intensity in different people based on personality (Cohen et al., 2007).

Personality, (e.g., individual variations in persons Fowers et al., 2023), is one of the key contributors to individual differences in stress responses (Bowling et al., 2005), including both perception of stressors and stress reactivity (Cohen et al., 2007). Among the personality theories tested in the last several decades, the Five Factor Model of personality (i.e., The Big Five; FFM) produces personality profiles that are significantly correlated with PS (Kotov et al., 2010; Lou and Li, 2023; Ringwald et al., 2024; Williams and Carlson, 2024). FFM posits that personality is best characterized across five domains, including neuroticism, extraversion, conscientiousness, agreeableness, and openness to experience (Costa and McCrae, 1992). FFM has been validated globally (McCrae and Costa, 1987), and neuroticism appears to have the strongest relationship with PS such that those high in neuroticism experience more stressors and perceive stress as more intense (Ringwald et al., 2024). Studies have also validated FFM’s ability to predict stress-related negative outcomes, for example low openness and the experience of PS appear to interact to worsen sleep (Williams and Carlson, 2024) and increase poor cardiac outcomes (Gallagher et al., 2018).

One may posit that the complex, heterogeneous nature of the PA-PS relationship may be in part related to differences in personality. For instance, it may be that those with higher neuroticism are more likely to experience PA as a stressor, while those higher in conscientiousness tend to experience the same stimulus as relaxing or enjoyable. Recent studies have suggested personality factors relate to both short-term and long-term PA engagement, as well as PA enjoyment (Caille et al., 2024; Dominski et al., 2020; Dominski et al., 2021; Engels et al., 2022; Huang et al., 2024). Yet, to date, no study to our knowledge has explored the interaction between PA and personality on PS. This knowledge of individual differences impacting PA will be key in moving the field of stress management toward person-centered and precision medicine (Yardley and Campbell, 2020), an approach that encourages tailored treatments to the individual patient based on that patient’s individual differences. Thus, the purpose of this study is to determine if any FFM personality factor moderates the PA-PS relationship and if so in what direction. Based on the literature, we predict that neuroticism, extraversion, conscientiousness, and PA will predict PS. Given the paucity of the research, we did not have *a priori* hypotheses regarding interaction effects.

## Materials and methods

### Participants

A convenience sample was utilized, targeting a broad audience. To participate, participants had to be over the age of 18. Exclusion criteria included a history of any anxiety disorder, a current physical injury, a chronic illness that prevented engagement in exercise, and pregnancy. Informed consent was obtained from all participants prior to their participation. Participants were told the purpose of the study was to understand the connection between personality, exercise and stress and that the survey would take about 60 min. Participants were told they would not be compensated directly, but that those who voluntarily completed the survey would be allowed to choose between three non-profit organizations for the principal investigator to donate to on their behalf. Participants were also told no personal information would be collected and that their anonymous data would be stored in a confidential manner by the principal investigator (first author). The study was approved by the Institutional Review Board at Gallaudet University.

Only participants who provided informed consent, were eligible, and completed all questionnaires and provided responses that were within expectations (e.g., total minutes of exercise [not PA] per day were under 24 h) were included.^1^ Eligibility for the study was confirmed by eliminating participants that provided answers on the demographic questionnaire that would render them ineligible to participate (e.g., history of anxiety disorder). Timestamps were unavailable from the survey platform and therefore unusual completion times could not be excluded. However, each participant was assigned a unique responder ID, which prevented redundant submissions.

### Materials

The instruments below were included in an open survey on the Survey Monkey platform. Items were not randomized or alternated. Adaptive questioning and completion checks were not used, and items were displayed for one questionnaire at a time. Participants were not allowed to check their answers before submitting. Unique sit visitor, cookies, log file analysis, IP checks, and view rates were not made available by the survey platform.

#### Perceived Stress Scale (primary outcome)

The Perceived Stress Scale-10 (PSS-10) is one of the most widely used self-report measures, containing 10 questions about how much stress an individual has perceived in the last month (Cohen et al., 1983; Lee, 2012). The PSS-10 takes approximately 10 min to administer. Although the questionnaire has been published using a 14-item, 10-item and 4-item format, Lee (2012) reviewed the literature published on these measures and concluded that the psychometric properties were highest for the 10-item version which was used in the current study. Further, the PSS-10 demonstrated an average Cronbach’s alpha of 0.84 for reliability (Lee, 2012). Other studies have supported this conclusion and concluded that the PSS-10 is a valid measure with diverse populations, with a Cronbach’s alpha of 0.82 (Andreou et al., 2011; Roberti et al., 2006).

#### International Physical Activity Questionnaire (IPAQ; predictor)

To assess the amount of PA the participants engaged in during the last week, the International Physical Activity Questionnaire - Long Version (IPAQ-long) was used. The self-report measure consists of 27 questions that describe the duration, frequency, intensity, and type of PA one has participated in within the last week. Specifically, the person is asked to answer how many days per week, as well as hours and minutes per day they participated in the following categories of activities: Bicycling for Transportation, Walking for Transportation, Moderate Housework Inside, Vigorous Housework Outside, Moderate Housework Outside, Moderate Leisure Activity, Vigorous Leisure Activity, Walking Leisure Activity. Each response is converted into total minutes per week and then multiplied by a metabolic equivalent (MET) weighting to compute MET minutes per week, and then summed to obtain the individual’s total MET’s per week. The total METs can also be interpreted using categorical scores including low, moderate and high levels of PA (see text footnote 1). The administration time for this assessment was approximately 20 min.

Craig et al. (2003) assessed the reliability and validity of the IPAQ-long in 12 different countries and determined that it demonstrated a test–retest reliability rate of 0.8, a concurrent validity rate of 0.67 and a criterion validity rate of 0.33 (pooled rho). Therefore, these researchers determined that the IPAQ-long was as reliable and valid as most other self-report measures and could be used with diverse populations (Craig et al., 2003). Further, the American Heart Association supports the use of the IPAQ as a short recall PA questionnaire (Strath et al., 2013).

#### International Personality Item Pool-NEO- 120-item Version (predictor)

The International Personality Item Pool-NEO- 120-item Version (IPIP-NEO-120) is a measure that was developed to mirror the NEO- Personality Inventory- Revised (NEO-PI-R). Similar to the NEO-PI-R, the IPIP-NEO-120 is based on the FFM theory of personality (Costa and McCrae, 2008; Johnson, 2014). This questionnaire measures personality using five factors, namely neuroticism, extraversion, openness to experience, agreeableness and conscientiousness. In addition, the measure also provides scores for six subscales associated with each main factor, resulting in scores on 30 dimensions. The test requires approximately 20 min to complete and is provided in a multiple-choice format.

In a study based on more than 21,000 participants, Johnson (2014) found that the IPIP-NEO-120 correlated with the well-established NEO-PI-R with alpha levels ranging between 0.76 and 0.87 for each of the personality dimensions (Johnson, 2014). In addition, the alpha reliability coefficients for the IPIP-NEO-120 ranging from 0.81 to 0.90 were obtained in a sample of more than 610,000 participants (Johnson, 2014). Based on these findings, Johnson (2014) concluded that the IPIP-NEO-120 is a valid and reliable assessment of personality.

### Procedures

Participants were recruited by listing the open survey link from the Survey Monkey platform on the Amazon Mechanical Turk website. The survey link was also listed on flyers posted at two universities and throughout two communities within the Mid-Atlantic region of the United States. The electronic survey was tested for usability and technical functionality prior to its use in this study and included validated instruments described above. The survey was voluntary and only items related to informed consent were required. Once the participant clicked the link to the online survey, they were asked to fill out screening questions that determined their suitability for the current study. If they did not meet criteria, a short explanation appeared on the screen stating that their answers indicated a lack of fit with the current study. If, however, they did meet criteria based on the screening questionnaires, the informed consent form appeared. The participant indicated their agreement to participate by selecting the “I agree” button at the bottom of the online consent form. The participant then had access to the aforementioned questionnaires. Data were collected in early 2017.

### Analysis

Descriptive statistics were used to describe demographics. Visual inspections of histograms were used to investigate normality. Given the non-normal distribution of PA scores, Spearman correlations between all variables were also conducted to determine the relationships. To investigate the extent to which PA and personality factors predict PS on their own or in combination, we fit a series of linear regression models. First, we fit separate models for each key predictor (i.e., single-predictor models). Next, we explored for interactions between each personality factor and PA in separate models. Finally, we fit a model including all main effects and interactions with *p* < 0.10, retaining any main effects that are part of an interaction. To aid in interpretation of any interaction effects, we inspected predicted PS scores for model individuals with high (i.e., one standard deviation above the mean) and low (i.e., one standard deviation below them) values for each variable in the interaction, and values for all other variables in the model equal to the mean for the sample. Visual inspection of the residual histogram was used to determine normality of residuals in the final model, and investigation of correlations and variance inflation factor was used to inspect for multicollinearity. Significance was established at *p* < 0.05. All analyses were conducted in SPSS version 29 (IBM Corp., Armonk, NY).

## Results

Six hundred thirty-six participants were screened for eligibility in the current study. Of those, 595 consented to participate and self-reported being eligible for participation. Another 56 were removed after validation checks deemed them ineligible due to age and self-reported history of an anxiety disorder. Two hundred sixty-three participants were missing data. Those who completed surveys showed very high conscientiousness scores which may have contributed to their decision to complete the surveys. Statistical comparisons between groups were not possible since those who did not complete the surveys did not have personality data. Taken together, these data did not appear to be missing at random and the use of multiple imputation was precluded. These participants were excluded from this study, resulting in a final sample of 276.

In total, 47% of participants were female, 38% were White, 26% were Asian American, 6% were Black, and 6% were Hispanic/Latino, with the majority of participants reporting regular engagement in, enjoyment of, and motivation to engage in exercise (see Tables 1, 2). Ninety three percent of the sample reported at least some college education and 75.4% reported participating in high school sports. Fourteen percent reported being diagnosed with a mental illness other than anxiety and nearly half (48.2%) reported experiencing an event that caused more PS than normal in the last month.

**Table 1:** Demographic information (*N* = 276).

Characteristic	%
Gender	
Male	53.3
Female	46.0
Other	0.4
Race	
Caucasian/White	38.4
Black/African American	6.2
Hispanic/Latino	5.8
Native American/Alaskan Native	1.1
Asian American	26.1
Other	20.3
Marital status	
Married	45.7
Divorced	3.3
Never married	40.6
Widowed	0.7
Separated	1.8
Living with someone	8.0
Education level	
Some high school	0.4
Graduated high school	6.5
Some college	21.0
Graduated college (4-year degree)	42.4
Some graduate school	6.2
Completed graduate school	23.6
Physical activity history	
Engaged in physical activity on a regular basis in the last month (% yes)	80.8
Motivated to exercise	
Not motivated /not very motivated	41.7
Somewhat motivated	39.1
Very motivated	15.2
Exercise Enjoyment	
Do not enjoy/do not enjoy very much	50.4
Somewhat enjoy	47.8
Participated in high school sports (% yes)	75.4
Participated in college sports (% yes)	47.5
Mental health history	
Diagnosed with mental illness (% yes)	14.9
Depression	9.8
Other mood disorder	2.1
Psychotic disorder	0.7
Developmental disorder	1.8
Eating disorder	2.2
Substance use disorder	1.1
Trauma related disorder	0.4
Other	1.5
Unsure	2.9
Seen by a mental health professional (% yes)	36.6
Experienced a stressful event in the last month that caused more stress than normal (% yes)	48.2

**Table 2:** Mean scores for perceived stress, self-reported physical activity, and big five personality traits (*N* = 276).

Measure	Mean	SD
PSS-10	17.01	6.74
IPAQ-10 Total MET	2994.81	2620.92
IPIP-NEO Neuroticism	65.13	16.2
IPIP-NEO Conscientiousness	87.28	15.36
IPIP-NEO Extraversion	76.5	13.45
IPIP-NEO Agreeableness	85.27	14.33
IPIP-NEO Openness to Experience	79.91	12.11

**Figure 1:**
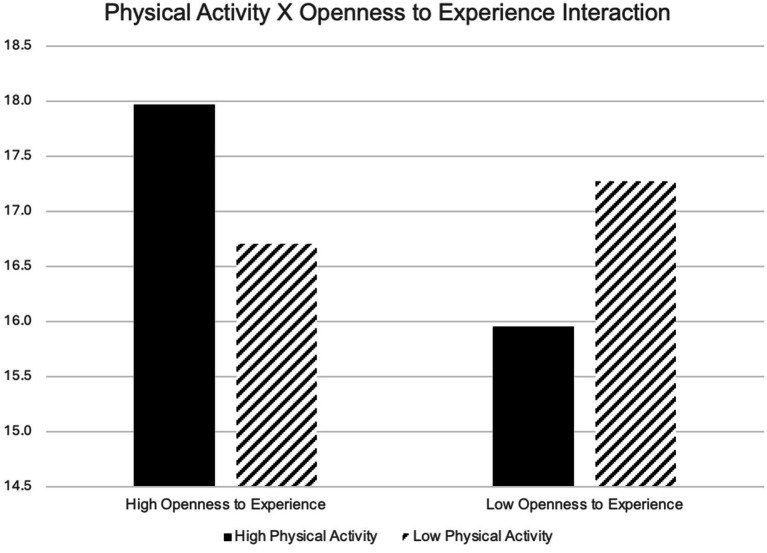
Depiction of the physical activity x openness to experience interaction. Graph depicts predicted stress scores; high values represent 1 SD above the mean, low values represent 1 SD below the mean.

Prior to conducting regression modeling, visual inspection confirmed the assumption of normal residuals, and correlations and variance inflation factor did not suggest issues with collinearity. In single predictor models, neuroticism (*B* = 0.259, *p* < 0.001) explained the most variance in PS, followed by conscientiousness (*B* = −0.191, *p* < 0.001), extraversion (*B* = −0.137, *p* < 0.001), and agreeableness (*B* = −0.099, *p* < 0.001). PA and openness to experience did not significantly predict PS. In simple interaction models, only openness to experience X PA was significant (*B* = 3.35, *p* = 0.014) suggesting that among those with high openness scores, more PA was associated with higher PS. The opposite was true among those with low openness scores (see Figure 1). The openness to experience X PA interaction was somewhat mitigated in a final model (see Tables 3, 4), such that the pattern remained consistent but was no longer statistically significant (*p* = 0.066). In this final model, conscientiousness was negatively associated with PS (*B* = 0.087, *p* < 0.001) and neuroticism was positively associated with PS (*B* = 0.221, *p* < 0.001). Agreeableness was not retained in the final model.

**Table 3:** Spearman’s correlations perceived stress, self-reported physical activity, and big five personality traits (*N* = 276).

Measure	PSS-10	IPAQ Total MET	IPIP-NEO-E	IPIP-NEO-A	IPIP-NEO-C	IPIP-NEO-N	IPIP-NEO-O
PSS-10	1.00	−0.11	−0.24***	−0.24***	−0.45***	0.63***	−0.07
IPAQ Total MET	−0.11	1.00	0.13*	0.07	0.11	−0.10	0.04
IPIP-NEO Extraversion (IPIP-NEO-E)	−0.24***	0.13*	1.00	0.10	0.29***	−0.24***	0.29***
IPIP-NEO Agreeableness (IPIP-NEO-A)	−0.24***	0.07	0.10	1.00	0.57***	−0.34***	0.43***
IPIP-NEO Conscientiousness (IPIP-NEO-C)	−0.45***	0.11	0.29***	0.57***	1.00	−0.54***	0.36***
IPIP-NEO Neuroticism (IPIP-NEO-N)	0.63***	−0.10	−0.24***	−0.34***	−0.54***	1.00	−0.12*
IPIP-NEO Openness to Experience (IPIP-NEO-O)	−0.07	0.04	0.29***	0.43***	0.36***	−0.12*	1.00

*p < 0.05, *** *p* ≥ 0.001.

**Table 4:** Final model determining the moderating relationship between personality, exercise behavior and perceived stress (*n* = 276).

Variable	*B*	SE B	β	*p*
Constant	16.166	3.308		<0.001
Extraversion	−0.047	0.026	−0.093	0.078
Conscientiousness	−0.087	0.026	−0.199	<0.001
Neuroticism	0.221	0.021	0.531	<0.001
Openness to Experience	−0.030	0.044	−0.053	0.506
Physical Activity	−0.002	0.001	−0.63	0.065
Physical Activity x Openness to Experience	2.02	0.000	0.632	0.066

## Discussion

The current study expanded previous work on the relationship between the FFM model of personality, PS, and PA by exploring the relationship amongst these three constructs (i.e., personality, PA, and PS) in a diverse adult sample. The results revealed that neuroticism and conscientiousness predicted PS but not PA, which offered partial support for our hypothesis that personality and PA would predict PS. Thus, this study provides additional support for the relationship between aspects of the FFM model and PS. Notably, an interaction between PA and openness to experience was also observed, whereby those with relatively low levels of openness were more likely to demonstrate lower levels of PS when they were active and vice versa. This pattern was not significant in the final model. Still, further investigation—particularly leveraging more accurate and objective measures of PA—are warranted to determine whether individuals who are highly active and highly open may be in need of additional stress management support.

The lack of relationship between PA and PS in this sample was unusual in view of the literature to date connecting personality and PA (Wilson and Dishman, 2015), as well as PA and PS (Chauntry et al., 2022; Perchtold-Stefan et al., 2020). However, participants in this study reported the equivalence of nearly 1,000 min of moderate-intensity PA per week, or nearly seven times the current PA guideline recommendations. These data may suggest that the relationship amongst these variables is less marked in those who self-identify as frequently engaging in exercise (i.e., structured PA) as part of their daily active energy expenditure or those who are high in conscientiousness similar to those in this sample. This surprising result could also be due to the use of self-report measures of PA versus objective measures or the use of total METs rather than specific domains of PA. These findings might suggest the importance of assessing current levels PA and personality characteristics during treatment as this could inform the decision of including a PA component to the intervention. Finally, our study revealed a surprising interactive relationship between openness to experience and PA on PS that may be related to novelty seeking. Given the preliminary nature of these exploratory findings, these possible explanations should be investigated further in future studies that include a less educated and less active sample.

### Strengths and limitations

One limitation of the current study was the use of self-report measures to measure the PA (i.e., IPAQ-Long) given relatively low correlation with objective measures (Beagle et al., 2020) and frequent overreporting of PA on self-report measures (Prince et al., 2008). There are, however, several strengths to this approach, including the ability to discriminate between various domains of activity and to collect data on large samples. Future work would benefit from a combined approach, using self-report and objective measures.

One strength of this study was the racial diversity of the sample, whereas previous studies have mostly included college students (Dalton, 2022; Maher et al., 2021). This diverse patient population was made possible through mTurk, which also created some limitations. Many participants did not provide viable or complete data, which resulted in a highly conscientious sample that reported frequent engagement in exercise. Future studies may benefit from using briefer versions of study measures and targeted recruitment for sedentary individuals.

## Conclusion

Expanding the understanding of how to match patients to stress management techniques that they are more likely to enjoy and therefore adhere to may guide the field toward precision medicine and decrease the far-reaching negative impacts of chronic stress. Beyond stress reduction, physical therapy and structured exercise have been proposed as novel frameworks for suicide prevention and depression management, underscoring their broader mental health benefits (Kanani, 2025).

## Data Availability

The raw data supporting the conclusions of this article will be made available by the authors, without undue reservation.

## Footnotes

^1^ https://sites.google.com/view/ipaq/score
